# Zinc-Containing Effluent Treatment Using *Shewanella xiamenensis* Biofilm Formed on Zeolite

**DOI:** 10.3390/ma14071760

**Published:** 2021-04-02

**Authors:** Inga Zinicovscaia, Nikita Yushin, Dmitrii Grozdov, Daler Abdusamadzoda, Alexey Safonov, Elena Rodlovskaya

**Affiliations:** 1 Department of Nuclear Physics, Joint Institute for Nuclear Research, Joliot-Curie Str., 6, 1419890 Dubna, Russia; ynik_62@mail.ru (N.Y.); grozdov@jinr.ru (D.G.); martinez-91@mail.ru (D.A.); 2 Department of Nuclear Physics, Horia Hulubei National Institute for R&D in Physics and Nuclear Engineering, 30 Reactorului, MG-6, 077125 Bucharest-Magurele, Romania; 3 Department of Biotechnology and Radioecology, Frumkin Institute of Physical Chemistry, Russian Academy of Science, 31 Leninsky Prospect, GSP-1, 119071 Moscow, Russia; alexeysafonof@gmail.com; 4 Laboratory for Heterochain Polymers, A.N. Nesmeyanov Institute of Organoelement Compounds of Russian Academy of Sciences, Vavilova Str., 28, 119991 Moscow, Russia; ro745@mail.ru

**Keywords:** hybrid sorbent, zeolite, industrial effluent, remediation, *Shewanella xiamenensis*

## Abstract

The sorption properties of *Shewanella xiamenensis* biofilm formed on zeolite (mineral-organic sorbent) as a sorbent have been investigated aiming to determine its suitability for complex zinc-containing effluent treatment. The optimum conditions for metal sorption from synthetic solutions were evaluated by changing the pH, zinc concentration, temperature, and time of sorption. The highest removal of metal ions was attained at pH range 3.0–6.0 within 60–150 min of sorbent-sorbate contact. The results obtained from the equilibrium studies were described using the Langmuir, Freundlich, and Temkin models. Maximum sorption capacity of the sorbent calculated from the Langmuir model changed from 3.4 to 6.5 mg/g. High coefficient of determination values calculated for pseudo-second-order and Elovich models indicate the predominant role of chemisorption in metal removal. Gibbs energy and ∆*H*° values point at the spontaneous and endothermic character of the sorption. The effect of pH and biosorbent mass on Zn(II) sorption from industrial effluent with an initial Zn(II) concentration of 52.8 mg/L was tested. Maximum removal of zinc ions (85%) was achieved at pH 6.0 by applying a two-step treatment system.

## 1. Introduction

The emission of toxic elements in the environment presents a serious risk for the ecological systems and human health, due to the nonbiodegradability of metals and their tendency to accumulate in living organisms, causing different diseases [1]. Therefore, reduction of the concentration of metal ions released in the environment is a priority task in order to prevent natural water pollution [2].

Zinc, being a vital component of more than 300 metal-enzymes and metal-proteins, is considered a critical element for the growth and development of living organisms [3,4]. It performs several important functions in organisms: structural, catalytic, and protective [5]. However, the release of zinc into water bodies in high concentrations may induce oxidative stress, damage of DNA molecules, and even growth and reproduction impairment [4]. Wastewaters containing zinc, among other toxic elements, are generated by mining activity, metallurgy and galvanizing industry, chemical industry, pharmaceutical industry, production of pigments, and pesticides [3,4,6].

Recently, a wide range of technologies has become available for wastewater treatment, among them chemical precipitation, flocculation, filtration, ion-exchange, coagulation, electrochemical treatment, membrane separation, oxidation-reduction, and adsorption [1,7,8]. However, these technologies are often low in terms of energy efficiency, as chemical product consumption is high, especially at metal concentrations of less than 100 mg/L, and some generate large volumes of sludge [7,8].

Biotechnological processes, in particular biosorption, have been described as a cost-effective technique to reduce the level of contaminants in wastewater without the need for toxic chemical compounds [2]. Among microorganisms, bacteria are of great interest for wastewater treatment since they have the ability to capture and remove pollutants from the environment related to the availability of a large number of functional groups on the cell wall [7]. The bacteria of the genus *Shewanella* are metal-reducing Gram-negative bacteria that are widely distributed and have been isolated from various environments [9]. *Shewanella* species can directly reduce uranium, chromium, iron, and manganese from the dissolved liquid state to insoluble oxides [10,11]. Bacteria of the genus *Shewanella* were tested as biosorbents in the studies by Mamba et al. [12] and Zinicovscaia et al. [13].

The main drawback of bacteria application in wastewater treatment is associated with the formation of a biofilm, meaning that the accumulation capacity is not constant and removal from the treated effluent is a difficult task [11,14]. The immobilization of microorganisms on a solid matrix is a technique that helps to circumvent this drawback. Zeolite is one of the materials widely applied as support for biofilm due to its high availability, stability, and excellent sorption properties, which ensure a high rate of metal ions’ removal [7,15]. Another advantage of zeolite is easy surface modification [1,15]. It is suggested that biofilm attachment to the support surface takes place through the production of extracellular polymeric substances, which contain polysaccharides, proteins, glycoproteins, DNA oligomers, and phospholipids, and act as a glue [8].

Information about Zn(II) removal using microbial biofilm formed onto a support material is very limited. The removal capacity of *Escherichia coli* and *Staphylococcus epidermidis* biofilms supported on kaolin toward zinc ions was investigated in a study by Quiton et al. [7]. *Escherichia coli* biofilm formed on zeolite was used for copper and zinc recovery from batch systems and real wastewater [14]. The capability of yeast (*Candida rugosa* and *Cryptococcus laurentii*) biofilm onto gravels to remove zinc from the batch system and real effluent was tested by Basak et al. [8].

In this study, the sorption capacity of *Shewanella xiamenensis* biofilm formed on zeolite (mineral-organic sorbent) for metal sorption from complex synthetic solutions and industrial effluent was examined.

To evaluate the removal efficiency, the effects of pH, zinc concentration, contact time, and temperature, on metal removal from synthetic solutions were studied. The equilibrium sorption isotherms and kinetics were applied for the description of experimentally obtained data. The efficiency of the *Shewanella xiamenensis* biofilm formed on zeolite for purification of industrial effluent depending on the pH of the effluent and sorbent dosage was studied as well.

## 2. Materials and Methods

### 2.1. Effluents

Four synthetic solutions and one industrial effluent containing zinc and Zn-accompanying metal ions were used in the present study (Table 1). The synthetic effluents were prepared using the chemicals purchased from Sigma-Aldrich (Darmstadt, Germany), which were of analytical grade. The zinc-containing effluent with pH 6.0 was obtained from an electroplating company (Atom, Dubna, Russia).

### 2.2. Preparation of Biosorbent

The zeolite (Supplementary Figure S1) used in the present study was obtained from the Chola deposit (Chita Region, Russia) and had the following composition: clinoptilolite (65.2%), cancrinite (1.5%), heulandite (Na) (22.7%), and heulandite (Ca) (12.1%). The main characteristics of the zeolite are: porosity—29.4–50%, surface area—22.3 m^2^/g, pore volume—0.19 cc/g, pore diameter—4.655 nm. Before analysis, zeolite was crushed in an agate mortar, sieved through 100 and 300 μm sieves, and dried at 100 ± 5 °C for 24 h.

Bacteria *Shewanella xiamenensis* DCB2-1 were provided by the Frumkin Institute of Physical Chemistry, Russian Academy of Science (Moscow, Russia). A detailed description of the strain can be found in Reference [16].

The bacteria were grown in the Adkins medium (Frumkin Institute of Physical Chemistry, Russian Academy of Science, Moscow, Russia) with 1.5 g/L of K_2_HPO_4_, 0.75 g/L of KH_2_PO_4_, 0.3 g/L of NH_4_Cl, 5.0 g/L of NaCl, 0.1 g/L of MgSO_4_·7H_2_O, 0.1 g/L of KCl, and 0.02 g/L of CaCl_2_ (pH 7.0). The medium was sterilized at 121 °C for 30 min, cooled to room temperature, inoculated with bacteria, and kept at 22 °C for 2 days. On the third day, 50 g of zeolite was introduced to the 250 mL of inoculum, and the biomass was grown until the seventh day. During the experiment, the pH of the cultivation medium was maintained constant by the addition of sterilized 0.1 M HCl (Sigma-Aldrich, Darmstadt, Germany) or NaOH (Sigma-Aldrich, Darmstadt, Germany). After 7 days, the zeolite with formed biomass was separated from the cultivation medium by filtration using a 5–8 µm “White Ribbon Filter” by Sigma-Aldrich (Darmstadt, Germany), freeze-dried (ScanVac CoolSafe, LaboGene, Frederiksborg, Denmark), and used for further experiments. Then, the sorbent was dried, homogenized, and packed in aluminum cups (JINR, Dubna, Russia) for neutron-activation analysis.

### 2.3. Biosorption Experiments

In the experiments with synthetic solutions, 0.5 g of sorbent and 50 mL of effluent were used. The pH of the solutions changed in the range 2.0–6.0, zinc concentration from 10 to 100 mg/L, contact time from 15 to 180 min, and temperature from 20 to 50 °C. The pH of the solutions was adjusted using NaOH or HNO_3_ (Sigma-Aldrich, Darmstadt, Germany). In order to assess the contribution of raw zeolite in metal removal, 0.5 g of zeolite were added to synthetic solutions of volume 50 mL (see Table 1) at pH 6.0, and samples were withdrawn at 15, 30, 45, 60, 90, 120, 150, and 180 min. All experiments were performed in duplicate at continuous agitation at 200 rpm (Reax 2, Heidolph, Schwabach, Germany) and room temperature (except thermodynamic studies).

In case of industrial effluent, to assess the effect of pH on metal sorption, it was changed from 2.0 and 6.0. The effect of the mass of sorbent on metal sorption was a two-stage process. Firstly, sorbent in the dosages 0.5–2.0 g/L was introduced to 100 mL of effluent. After 120 min, the sorbent was removed by filtration and a new amount of sorbent (0.5 g) was added to effluents obtained after the first stage of treatment. The time of sorbent interaction with sorbate during the second stage was 120 min. Then, the sorbent was removed from the solution using a 5–8 µm “White Ribbon Filter” by Sigma-Aldrich (Darmstadt, Germany).

The content of metal ions sorbed by sorbent *q* (mg/g) was calculated using Equation (1):

(1) $$q=\frac{V\left(C_{i}-C_{f}\right)}{m}$$

and efficiency of metal removal, *E* (%), using Equation (2):

(2) $$E=\frac{C_{i}-C_{f}}{C_{i}}\times 100$$

where *V* is the volume of the solution in mL, *C_i_* and C*_f_* are the initial and final metal concentrations, mg/L, and *m* is the mass of sorbent, g.

### 2.4. Methods

The efficiency of metal sorption in batch experiments was determined by means of neutron-activation analysis (NAA) (JINR, Dubna, Russia). NAA is a highly sensitive and reliable analytical technique, which allows the determination of a wide range of elements using small samples and without any chemical pre-treatment, unlike ICP-MS/ICP-AES and other techniques. The method is nondestructive, based upon the conversion of stable isotopes of chemical elements to unstable radioactive isotopes by irradiation with neutrons at a nuclear reactor [17].

Samples were irradiated for 72 h with epithermal neutrons, repacked, and measured twice. The analysis of the spectra and calculation of metal concentrations was done using the Genie2000 software (2000, Canberra, Meriden, CT, USA) and “Concentration” (Version 9, JINR, Dubna, Russia) software. Quality control of the measurements was ensured by reference materials irradiated simultaneously with the samples: 1633c—Trace Elements in Coal Fly Ash (National Institute of Standards and Technology, Gaithersburg, MD, USA), BCR-667—Estuarine Sediment (Joint Research Centre, Brussels, Belgium), and CTA-FFA-1—Fine Fly Ash (Institute of Nuclear Chemistry and Technology, Warsaw, Poland). The difference between obtained and certified values was within 2–10%.

Copper in model solutions was determined in accordance with the procedure presented in our previous studies [18,19]. Metal concentrations in the effluent were determined using ICP-MS systems (the Element 2™, the Thermo Scientific, Chemnitz, Germany) [20].

**Figure 1:**
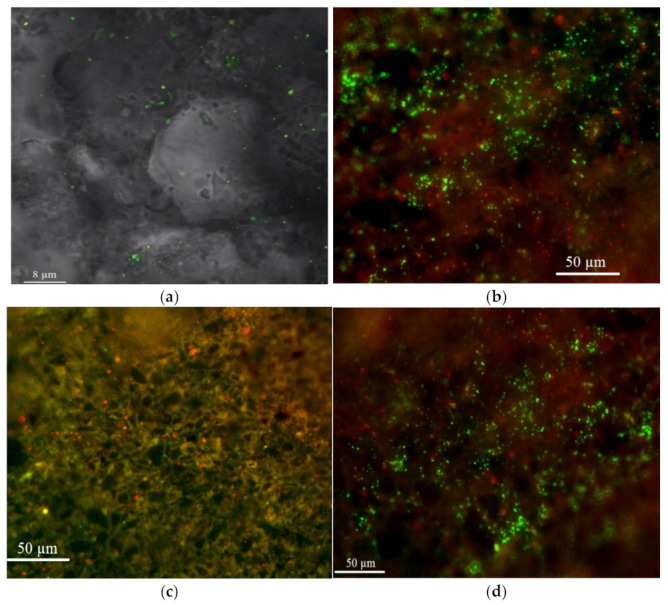
Zeolite surface (**a**) raw and (**b**–**d**) modified (polysaccharides are red bacterial cells and DNA is green).

A laser confocal scanning microscope (Leica SP5, Berlin, Germany) allowed visualization of the biofilm formed on zeolite. Polysaccharide matrix was stained with lectin IV from wheat germ agglutinin (WGA, Sigma-Aldrich, Darmstadt, Germany) conjugated with fluorescent dye Alexa Fluor 488 (W11261 ThermoFisher, Waltham, MA, USA). For cell visualization, fluorescent dye SYTO^®^ 11 (S7573 ThermoFisher, Waltham, MA USA) diluted 1:1000 in phosphate buffer (Sigma-Aldrich, Darmstadt, Germany) was applied. An argon laser with a wavelength of 488 nm was used for detecting WGA fluorescence and of 594 nm for detecting SYTO 11. The Nomarski contrast method for applied identification of undyed particles (Figure 1) was used. The Imaris software package (Version 7.0.0, Bitplane, Zurich, Switzerland) was applied to determine the area occupied by bacterial cells and polysaccharide matrix.

Infrared spectra were obtained employing the Nicolet 6700 spectrometer (Thermo Scientific, Waltham, MA, USA) using a zinc selenide ATR crystal. Spectra were recorded at a spectral resolution of 2 cm^−1^, averaging 48 scans of each sample in total internal reflection mode. Samples were analyzed without any preliminary pretreatment.

The specific area of the mineral-organic sorbent was calculated using the BET (Brunauer–Emmett–Teller) equation. Pore size distribution was derived by applying the density function theory (DFT) [11]. Zeta potential of the prepared sorbent was measured using a Zetasizer Nano ZSP (Malvern Instruments, Malvern, UK). To determine the thermal stability of raw and modified zeolites, thermal gravimetric analysis (TGA, NETZSCH TG 209 F1 Libra TGA209F1E-0199-L, Selb, Germany) was performed.

## 3. Results

### 3.1. Sorbent Description

Images obtained by the confocal laser scanning microscope showed that biofilm covered 81.6% of the surface of zeolite and it consists of bacterial cells (7.3%) and polysaccharides (74.3%), and 18.4% of the surface remained uncovered (Figure 1). A small number of bacteria were detected on the raw zeolite as well.

The specific surface areas of the zeolite covered by biofilm calculated from the BET equation constituted 34 m²/g. The total volume of pores was 0.154 cm^3^/g, with dominant pore sizes of 3.0, 5.5, and 7.5 nm. The zeta potential of the prepared sorbent was shown to be negative at the measured pH range of 2.0–6.0.

Using NAA, it was possible to determine 24 elements in the raw zeolite, while in modified zeolite, 23 elements were detected (Table 2). The concentration of Mg, Cl, and Ca in modified zeolite increased in comparison with raw zeolite, while for Cs and Gd it was below the method detection limits. The concentrations of other elements in raw and modified sorbent were similar.

**Figure 2:**
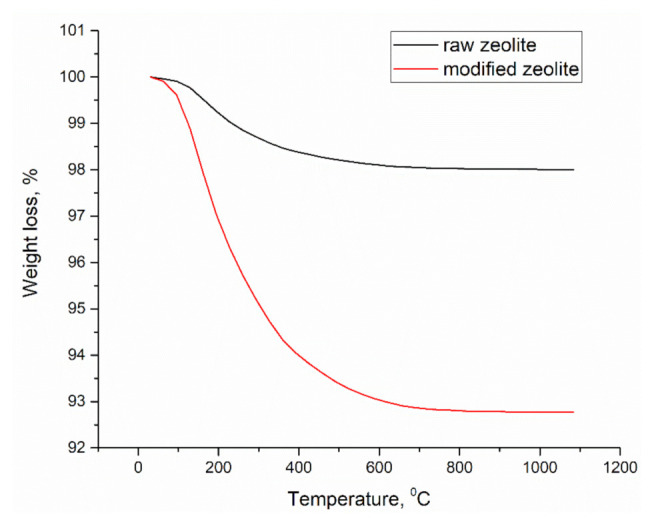
Thermal gravimetric analysis (TGA) diagram of raw zeolite and zeolite with biofilm.

Comparative TGA curves of raw and modified zeolite are presented in Figure 2. From the obtained thermographs, it is seen that for raw zeolite with the increase of temperature up to 1083 °C, the percentage of weight loss was only 2%, and for zeolite with a biofilm, −7.2%.

### 3.2. Sorption of Metal Ions from Synthetic Solutions

#### 3.2.1. Influence of pH on the Removal of Metal Ions from Synthetic Solutions

**Figure 3:**
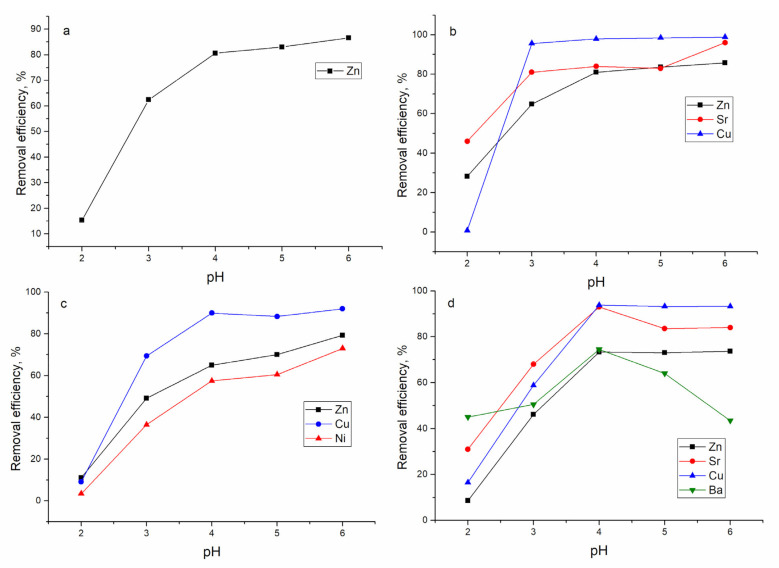
Effect of pH on metal removal from studied synthetic solutions by mineral-organic sorbent: (**a**) Zn, (**b**) Zn/Sr/Cu, (**c**) Zn/Ni/Cu, and (**d**) Zn/Sr/Cu/Ba systems.

The pH is a parameter that significantly affects the metal removal process. The optimal pH for maximum metal removal, as seen in Figure 3, changed from 3.0 to 6.0. The pH was chosen in the range of 2.0–6.0 due to the formation of precipitation above pH 7 [21]. In the Zn-system, removal of zinc ions increased with the pH increase and maximum removal (86.6%) was attained at pH 6.0. Zinc removal by *Escherichia coli* and *Staphylococcus epidermidis* biofilms supported on kaolin was carried out at pH 5.0 [7].

In the Zn/Sr/Cu system, the increase in pH also promoted metal ion removal. The highest zinc and strontium removal were achieved at pH 6.0: 85.8% and 96%, respectively. The sorption of copper ions was favorable at pH 4.0–6.0 when 98% of removal was achieved.

In the Zn/Ni/Cu system, as in previously described systems, increase of pH facilitates metal ions removal, and maximum values for all metal ions were achieved at pH 6.0: 79.3% for Zn(II), 92% for Cu(II), and 73% for Ni(II). In the four-component system, Zn/Sr/Cu/Ba, pH 4.0 was optimal for strontium and barium removal, 93% and 74.5%, respectively. Maximum Zn(II) and Cu(II) removal were achieved at pH 4.0 and remained constant over the pH range 4.0–6.0. The maximum removal efficiencies of *Escherichia coli* biofilm supported on zeolite were obtained in pH values of 4.8–5.7 and 4.5–5.5 for copper and zinc, respectively [14]. At pH 6.0, maximum removal of Zn(II) by the yeast biofilm was attained, where 44% of Zn(II) was removed by *Candida rugosa* biofilm and 37% by *Cryptococcus laurentii* biofilm [8].

The low removal efficiency of metals present in the analyzed systems at acidic pH is explained by their competition with protons for binding sites [14]. With increasing pH, more negatively charged functional groups are available, resulting in greater attraction for the cationic species [8]. In the analyzed systems at pH 2.0–6.0, metal ions are present in the solution in cationic form. Further experiments were performed at pH 6.0.

#### 3.2.2. Influence of Time on Metal Sorption

Studying the effect of time on metal ion removal showed that biosorption of metal ions by mineral-organic sorbent is a relatively fast process, and equilibrium was attained within 60–150 min, which indicates that chemical sorption (ion-exchange, metal binding to functional groups) contributes mainly to the removal of metal ions by the studied sorbents.

Holub and Balintova [15] studying Zn and Cu sorption by zeolite have shown that equilibrium was established in 4 h. The equilibrium times in sorption of copper and zinc onto *Escherichia coli* biofilm formed on zeolite were obtained after 5 and 4 days, respectively [14]. In the binary (Cu-Zn) system, 41% of Cu and 16% of Zn were removed from the aqueous solution by hydroxyapatite with an equilibrium time of 12 and 9 h, respectively [22].

**Figure 4:**
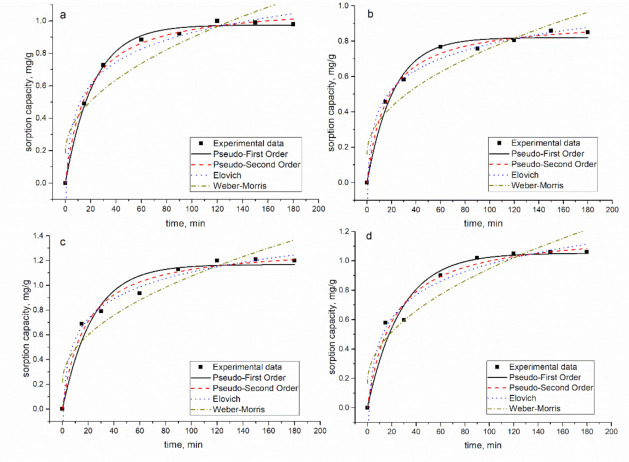
The kinetic curves for Zn ion sorption on mineral-organic sorbent: (**a**) Zn, (**b**) Zn/Sr/Cu, (**c**) Zn/Ni/Cu, and (**d**) Zn/Sr/Cu/Ba systems.

In the Zn-system, 100% of zinc ions were removed from the solution in two hours of sorbent-sorbate interaction (Figure 4 and Supplementary Figure S2). In the case of experiments with raw zeolite, 74% of Zn(II) were removed from the solution (Supplementary Figure S3). In the Zn/Sr/Cu system, for maximum zinc ions removal, 85% was attained in 120 min. Maximum copper and strontium removal, 95% and 44% respectively, was reached in 150 min. Using raw zeolite, 60% of Zn(II), 20% of Sr(II), and 70%of Cu(II) were removed from solution (Supplementary Figure S3). In the Zn/Ni/Cu system, the equilibrium for zinc ions with maximum metal ion removal of 90% was achieved in 120 min. For Ni(II) and Cu(II), the optimal time for maximum metal ion removal was 80 and 150 min, respectively. The efficiency of metal ion removal by raw zeolite was lower and constituted 68% for Zn(II), 63% for Ni(II), and 54% for copper ions.

In the Zn/Sr/Cu/Ba system, for Zn(II), Sr(II), and Cu(II), equilibrium was established in 150 min. Metal removal at equilibrium constituted 86% for Zn(II), 73% for Sr(II), and 86% for Cu(II). Ba(II) removal in the first 60 min of sorbent-sorbate interaction was on the level of 26%, then it significantly decreased with the increase of the time of contact, up to 4% (Supplementary Figure S2). The decrease of Ba(II) removal can be explained by the decrease in the number of binding sites of the sorbents and functional groups’ affinity for other metal ions present in the system. Using raw zeolite, 64% of Zn(II), 40% of Sr(II), 59% of Cu(II), and 10% of Ba(II) were removed from synthetic solutions (Supplementary Figure S3). Thus, the metal removal efficiency of the mineral-organic sorbent for Zn(II), Sr(II), and Cu(II) in analyzed systems was on average 21–27% higher than that of raw zeolite. Removal of Ni(II) by mineral-organic sorbent was by 35% higher than by raw zeolite, while removal of Ba(II) was higher by raw zeolite (by 6%).

The process of metal removal with respect to time can be divided into two stages: fast sorption, followed by a slow stage with equilibrium achievement. At the first stage, the sorption of metal ions is quick due to the abundance of binding sites. At this stage, the main mechanism of metal ion interaction with sorbent can be considered as ion exchange [15] and metal trapping to the functional groups of *Shewanella xiamenensis.* Lowering the sorption in the next stage can be associated with the decrease of binding sites, as well as the decrease of metal ion concentrations with time [1].

Zinc removal from the Zn-system was the highest and it was reduced in multi-metal systems, especially in the four-component system. Lower zinc removal from complex systems can be explained by the competitive sorption of heavy metals [22]. This is in agreement with the Salehizadeh and Shojaosadati [23] study, which showed that removal of copper, zinc, and lead from the multi-component system was lower than from single-element systems due to interactive and interference effects. The removal of copper and zinc by hydroxyapatite from the binary system was almost two times lower compared to the single-metal system [22].

Data obtained experimentally were fitted to four kinetic models: pseudo-first-order model (PFO), pseudo-second-order model (PSO), Elovich model (EM), and Weber and Morris intraparticle diffusion model (IPM). The description of the models can be found in References [13,19].

The graphical representation of the models along with experimental values for Zn(II) is presented in Figure 4, while for other metal ions, in Supplementary Figures S4–S6. The experimental and theoretically calculated sorption capacity values and coefficients related to kinetic plots for Zn(II) are given in Table 3, while for other metal ions, in Supplementary Table S1.

The experimentally obtained (*q_exp_*) and calculated values (*q_e_,_cal_*) for PFO and PSO models were very close, confirming that both models were suitable for describing the sorption kinetic data. The values of sorption obtained for the PSO were higher than for the PFO one, indicating a higher rate of sorption [13].

However, since the *R^2^* values for PFO, PSO, and EM models were relatively high (see Supplementary Materials, Table S1), the Akaike Information Criterion (AIC) test was applied to emphasize which model best describes the experimentally obtained values. According to the AIC test, the PSO model better describes Zn(II) biosorption in all systems. In the Zn/Cu/Sr system, the AIC test revealed the suitability of the PFO model to describe the data obtained for strontium ions, and of the EM for data obtained for Cu(II). EM fit well with the data presented for Cu(II) sorption in the Zn/Ni/Cu and Zn/Cu/Sr/Ba systems. The PSO model showed its suitability for the description of data obtained for Sr(II) in the Zn/Cu/Sr/Ba system.

Since PSO and EM better described the sorption data, it can be assumed that chemisorption is the rate-controlling mechanism for the biosorption [7]. Metal sorption from complex nickel-containing effluents onto *Shewanella xiamenensis* biofilm placed on zeolite was better described by PFO and PSO models [13].

#### 3.2.3. Influence of Zinc Concentration on Metal Sorption

**Figure 5:**
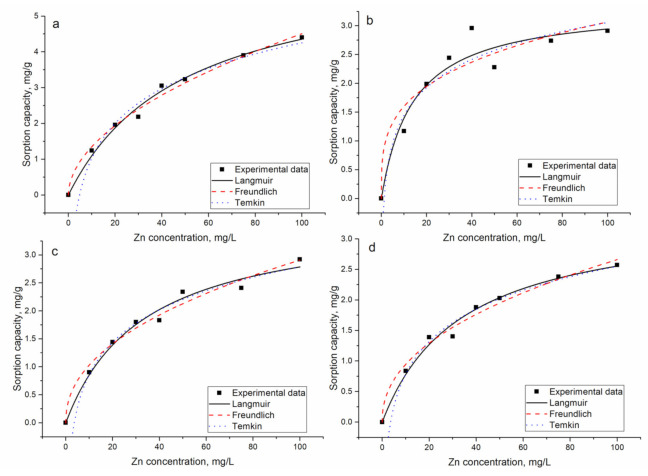
The sorption isotherms for Zn(II) ion removal by mineral-organic sorbent: (**a**) Zn, (**b**) Zn/Sr/Cu, (**c**) Zn/Ni/Cu, and (**d**) Zn/Sr/Cu/Ba systems.

The effect of initial zinc concentration on the sorption of metal ions present in analyzed systems was studied by varying it from 10 to 100 mg/L during 120 min. The concentration of other metal ions in the solution was maintained constant. It can be inferred from Figure 5 that the sorption capacity of the mineral-organic sorbent sharply increased with the increase of Zn(II) concentration in solution (Figure 5), from 1.2 to 4.4 mg/L in the Zn-system, from 1.2 to 2.9 in the Zn/Cu/Sr system, from 0.9 to 2.9 in the Zn/Ni/Cu system, and from 0.8 to 2.6 mg/g in the Zn/Cu/Sr/Ba system. At low metal ion concentrations, a large number of available binding sites facilitate metal ion sorption, while at high metal concentrations, the number of binding sites on sorbent surfaces is limited and a sorption maximum has been attained [24].

In the Zn/Cu/Sr system, the increase of Zn(II) concentration resulted in significant decrease of Sr(II) removal, from 54% at Zn(II) concentration 10 mg/L to 0.4% at concentration 100 mg/L. Copper removal was less affected by the rise of Zn(II) concentration and decreased only by 6%. In the Zn/Ni/Cu system, the increase of Zn(II) concentration in solution led to the decrease of Ni(II) removal by 60% and of Sr(II) by 20%. In the Zn/Cu/Sr/Ba system, the increase of Zn(II) concentration mainly affected Sr(II) and Ba(II) removal, resulting in the decrease of their removal by 45% and 40%, respectively. These results are in line with previous experimental data, where SynAllo-2 sorbent showed a preferable uptake of zinc ions over barium and strontium ions [24]. Removal of Cu(II) was reduced only by 15%. In Holub and Balintova’s [15] study, it was shown that copper ions are sorbed better on zeolite in comparison with zinc ions. However, since the experimental conditions in the mentioned work were different from the present study, it is difficult to compare the data.

Experimental data were fitted with the Langmuir, Freundlich, and Temkin isotherm models, presented in References [13,19].

Separation factors, *R_L_,* which predict the potential sorption probability relationship between solid and liquid, were calculated according to Equation (3):

(3) $$R_{L}=\frac{1}{1+bC_{0}}$$

The isotherm constants for Zn(II) presented in Table 3 and for other elements in Supplementary Table S2 were calculated by nonlinear regression (Figure 5). The applicability of the isotherm equations was determined by comparing the coefficients of determination (*R*^2^). The Langmuir and Freundlich isotherms describe the experimentally obtained data well for the Zn-system. In the Zn/Cu/Sr system, the experimental data were better fitted by the Langmuir model. In Zn/Ni/Cu, all applied models adequately described the data, while in the Zn/Cu/Sr/Ba system, the Langmuir and Temkin models were more applicable. The highest value of the maximum sorption capacity calculated from the Langmuir model was defined for the Zn-system, while in the multi-metal system, its values were very close and almost two times lower than in the Zn-system. However, the AIC test showed the applicability of the Langmuir model for the description of experimentally obtained data.

The *R_L_* lower than 1.0 indicates that metal ion sorption by mineral-organic sorbent was favorable for all metal ion concentrations [24,25]. Freundlich constant *n* value lying in the range of 1–10 for single and complex systems confirms the favorable conditions for sorption [22]. Also, since *n* values in the present study were higher than 1.0, chemical absorption can be considered dominant for Zn(II) ions’ sorption [26].

According to the Temkin isotherm constant (B) values, sorption can be considered a physical process, since the values were in the range 5–40 kJ/mol [27]. The applicability of several isotherm models to the biosorption of Zn(II) onto mineral-organic sorbent indicates the complex nature of sorption when both monolayer sorption and heterogeneous energetic distribution of active sites on the surface of the biosorbent occur [7].

Biosorption of zinc on *Escherichia coli* and *Staphylococcus epidermidis* biofilms supported on kaolin was best described by the Freundlich model [7]. The experimental data of biosorption of copper and zinc on *E.coli* placed on zeolite revealed better results with the Langmuir isotherm [14]. The comparison of the sorption capacity of the analyzed sorbent with data presented for other sorbents in the literature is summarized in Table 4.

#### 3.2.4. Influence of Temperature on Metal Sorption

**Figure 6:**
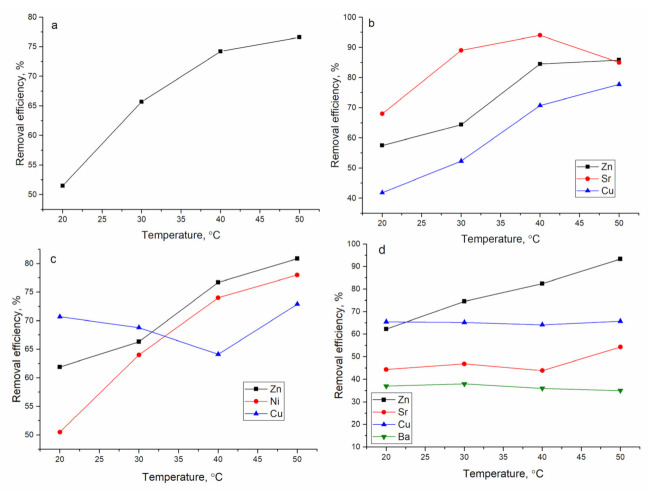
Effect of temperature on metal removal by mineral-organic sorbent (**a**) Zn, (**b**) Zn/Sr/Cu, (**c**) Zn/Ni/Cu, and (**d**) Zn/Sr/Cu/Ba systems.

The temperature of the solution is another important parameter which affects metal removal. High temperature enhances biosorption efficiency through increased surface activity and kinetic energy of the solute [29]. The increase of temperature in all systems increased Zn(II) removal at least by 20% (Figure 6). Thus, in the Zn-system, it increased from 51% at 20 °C to 77% at 50 °C, in the Zn/Cu/Sr system from 58% to 86%, in the Zn/Ni/Cu system from 62% to 81%, and in the Zn/Cu/Sr/Ba system from 62% to 93%. The increase in Zn(II) sorption can be associated with an increase in the mobility of the metal ions as a result of acquired energy in the system. This also indicated that Zn(II) biosorption is an endothermic process, which could also be considered as chemical sorption [25]. Removal of Cu(II) was influenced by temperature rise only in the Zn/Cu/Sr system, while in the other two systems, its removal was not influenced by temperature. In the Zn/Cu/Sr system, maximum removal of Sr(II), 94%, was achieved at a temperature of 50 °C. Ni(II) removal grew from 50% to 78% with temperature increase. In the Zn/Cu/Sr/Ba system, Sr(II) and Ba(II) removal were not influenced by temperature. High metal removal in complex systems can be explained by the synergetic effect of the biofilm that, after ion enrichment, allows transportation of the metal ions to deeper sites of the support through the exopolysaccharide net, liberating some external surface sites [30].

The Δ*G*°, Δ*H*°, and Δ*S*° were computed from the Equations presented in Reference [13]. The enthalpy and entropy values were calculated by a plot of ln*K_d_* versus 1/*T* (Supplementary Figure S7), and the results are presented in Table 3 for Zn(II), and for other elements, in Supplementary Table S3.

At all temperatures, the values of Δ*G*° were negative, indicating the feasibility of the process and spontaneous nature of the sorption [26]. The positive value of Δ*H*° calculated for all elements, except Cu(II) and Ba(II) in the Zn/Cu/Sr/Ba system, showed that the sorption process is endothermic. The positive value of Δ*S*° suggests increased randomness at the solid-solution interface during metal ion sorption on hybrid sorbent [26].

**Figure 7:**
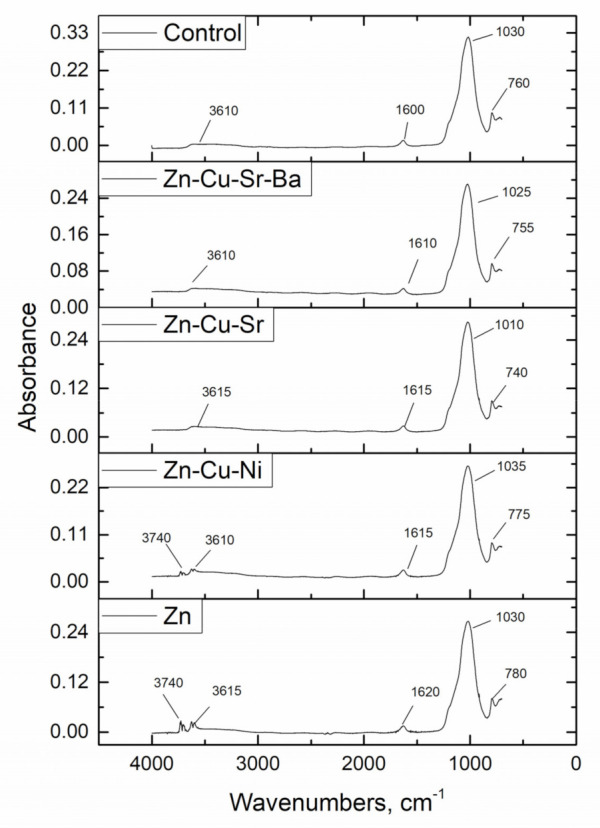
FTIR spectra of control and metal-loaded mineral-organic sorbents.

According to Figure 7, in the unloaded sorbent, the bands at 760 and 1030 cm^−1^ are attributed to O–Si–O and Si–O–Al groups [31]. Bands at wavenumber region 3610 cm^−1^ and deformation at area 1620 cm^−1^ could be assigned to –OH groups. The intensities of these bands were the highest in the control samples and that could be attributed to the hydrophilic nature of zeolite [32] and to –OH groups of the biofilm layer.

The absorption band positions in the IR spectrum of the Zn-system were shifted by 10 ÷ 20 cm^−1^, indicating the involvement of −OH and Si–O groups in Zn(II) binding. The splitting of the signal in the area of 3610 cm^−1^ on the initial band 3610 and 3740 cm^−1^ takes place due to the displacement of water from the zeolite. In the Zn/Cu/Sr system, shifting of the band positions by 20 cm^−1^ points to the participation of –Si–O– and Si–O–Si in metal ion sorption. In the Zn/Ni/Cu system, the splitting of bands in the area 3610 cm^−1^, similar to the Zn-system, indicates dominant Zn(II) sorption. For other functional groups, the shift of their position was by 5–7 cm^−1^. In the Zn/Cu/Sr/Ba system, a pattern similar to the Zn/Cu/Sr system was observed. One of the possible mechanisms of metal sorption can be considered the formation of metal bi- and mono-dentate ligands with OH groups and subsequent strong electrostatic interaction with charged sorbent surfaces [24].

### 3.3. Metal Sorption from Industrial Effluent

**Figure 8:**
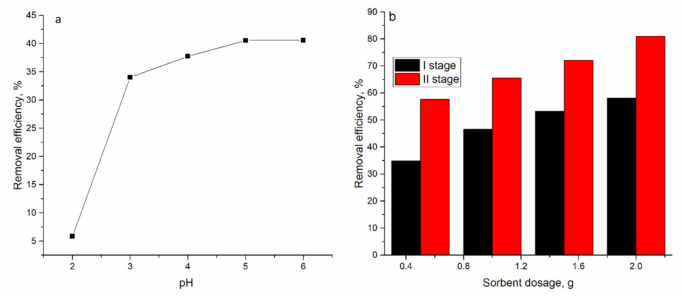
Effect of (**a**) pH and (**b**) sorbent dosage on Zn(II) removal from industrial effluent by mineral-organic sorbent.

The industrial effluent analyzed in the present study contained Zn(II) in a concentration of 52.8 mg/L, while concentrations of accompanying metal ions were significantly lower, and they are not discussed. The effect of two parameters, the pH of the effluent and sorbent dosage on the mineral-organic sorbent removal capacity, was investigated. The effect of time on the efficiency of metal removal was not assessed since in our previous study it was shown that the time required to achieve equilibrium in experiments with the model solution and real wastewater was the same [33]. The necessity of the study of the effect of sorbent dosage was determined by higher Zn(II) concentration in comparison with the synthetic solution. The pH of the effluent changed from 2.0 to 6.0. As in the case of the synthetic solution, the lowest efficiency of Zn(II) removal was obtained at pH 2.0 (5.7%) and it increased to 41% at pH 6.0 (Figure 8). Thus, initial effluent pH was shown to be optimal for Zn(II) ions’ removal. The next experiment was performed at pH 6.0, varying the dosage of sorbent from 0.5 to 2.0 g. An increase of sorbent dosage leads to a rise of Zn(II) ion removal by only 18% (from 40% to 58%). Relatively low Zn(II) removal at high sorbent mass can be associated with biosorbent particle agglomeration, which decreases its specific surface area. It became apparent that multiple sorption cycles are needed to decrease the Zn(II) concentration in the effluent. Thus, a new dosage of sorbent (0.5 g) was added to effluents obtained after the first cycle of treatment.

The efficiency of Zn(II) removal during the second stage varied from 35% (effluent treated with 0.5 g sorbent in the first stage) to 54% (effluent treated with 2.0 g sorbent in the first stage). Thus, by varying the sorbent dosage in two stages, it was possible to remove Zn(II) from the effluent from 58% to 85%.

*Escherichia coli* biofilm placed on zeolite removed copper and zinc cations with efficiencies of 51.28% and 48.25% respectively, from wastewater containing 53 mg/L of copper and 61 mg/L of zinc. In the case of wastewater containing 3.1 mg/L of copper and 2.3 mg/L of zinc, removal efficiencies constituted 94.75% and 92.96%, respectively [14]. *Candida rugosa* biofilm on gravel was able to remove up to 95% of Zn(II) from real effluent containing 85 mg/L of zinc ions, leaving 4 mg/L residual Zn(II) ion in the treated effluent [8].

The price of the sorbent is a critical parameter, which determines its wide application. The price of the produced sorbent is 580 USD per ton. The price of hybrid sorbents mainly depends on the cost of the support, which varies in a wide range, depending on the supplier. In our case, the price of the zeolite was 380 USD per ton. The price of salts required for the preparation of one liter of cultivation medium for *Shewanella xiamenensis* growth constituted 0.2 USD. The price of the studied sorbent is comparable with the price of sorbents studied by Ramesh et al. [22] and lower than the price of sorbents presented by Lu et al. [34].

## 4. Conclusions

The applicability of environmentally friendly mineral-organic sorbent for complex zinc-containing solutions and real effluent treatment was tested. The maximum uptake of metal ions from synthetic solutions occurred in 60–120 min at pH 6.0. The maximum sorption capacity of sorbent calculated according to the Langmuir model varied from 3.4 to 6.5 mg/g. The highest metal removal took place in the single-metal system. Pseudo-second-order and Elovich models better described the kinetic data, pointing out the predominant role of chemisorption in metal removal. The sorption can be characterized as a spontaneous, predominantly endothermic process. The initial pH of industrial effluent was found as optimal for maximum Zn(II) removal. Multiple sorption cycles are needed to attain high efficiency of zinc ions’ removal from the industrial effluent.

## Figures and Tables

**Table 1:** Chemical composition and metal concentrations in four analyzed synthetic solutions and real effluent.

	Concentration, mg/L				
**Synthetic Solutions**					
**System**	**Zn**	**Cu**	**Ni**	**Sr**	**Ba**
Zn	10 ± 0.3	-	-	-	-
Zn/Sr/Cu	10 ± 0.2	5 ± 0.04	-	1 ± 0.01	-
Zn/Ni/Cu	10 ± 0.3	2 ± 0.03	2 ± 0.02	-	-
Zn/Sr/Cu/Ba	10 ± 0.2	2 ± 0.06	2 ± 0.03	-	1 ± 0.02
**Industrial Effluent**					
Effluent	52.8 ± 0.8	0.06 ± 0.002	0.8 ± 0.02	0.34 ± 0.01	0.03 ± 0.001

**Table 2:** Elemental content of raw and modified zeolite as determined by neutron-activation analysis (NAA).

Element	Raw Zeolite	Modified Zeolite	Element	Raw Zeolite	Modified Zeolite
	Concentration, µg/g			Concentration, µg/g	
Na	13,000 ± 1000	8590 ± 500	Sr	66.2 ± 6	100 ± 8
Mg	6600 ± 600	7400 ± 800	Rb	217 ± 20	186 ± 15
Al	67,700 ± 1500	61,300 ± 3000	Sb	0.25 ± 0.01	0.27 ± 0.02
Si	260,000 ± 26,000	270,000 ± 19,000	Ba	128 ± 10	205 ± 20
Cl	<81	180 ± 25	Cs	7.57 ± 0.2	n.d.*
K	29,000 ± 2900	28,900 ± 3000	Ce	21.1 ± 2	15 ± 1.5
Sc	1.8 ± 0.06	1.9 ± 0.06	Eu	1.0 ± 0.08	0.6 ± 0.06
Ca	10,000 ± 800	18,000 ± 1800	Gd	3.6 ± 0.2	n.d.
Mn	350 ± 17	110 ± 7.5	Tb	0.4 ± 0.01	0.7 ± 0.03
Fe	8600 ± 600	7900 ± 500	Yb	2.1 ± 0.02	1.2 ± 0.01
Zn	60 ± 2.5	54 ± 2.0	Hf	6.3 ± 0.6	5.9 ± 0.04
Br	0.74 ± 0.02	0.98 ± 0.05	Th	17.1 ± 1.0	15.5 ± 0.7
La	12.5 ± 0.5	10 ± 0.6	-	-	-

*—not detected.

**Table 3:** Parameters determined for Zn(II) sorption on mineral-organic sorbent.

Model	Parameter	System			
		Zn	Zn/Cu/Sr	Zn/Ni/Cu	Zn/Cu/Sr/Ba
**Kinetic studies**					
PSO	*q_e,cal_*, mg/g	1.1	0.93	1.3	1.2
	*k*_2_, g/mg·min	0.05	0.06	0.04	0.02
	*R* ^2^	0.99	0.99	0.98	0.98
**Equilibrium Studies**					
Langmuir	*q_m_*, mg/g	6.5	3.4	3.7	3.4
	*b*, L/mg	0.02	0.04	0.03	0.03
	*R_L_*	0.3–0.8	0.1–0.6	0.3–0.8	0.2–0.8
	*R* ^2^	0.99	0.95	0.98	0.99
**Thermodynamic Studies**					
∆*G*°, kJ/mol	293 K	−9.7	−10.4	−9.9	−9.8
	303 K	−10.4	−10.6	−10.4	−10.6
	313 K	−11.1	−11.3	−11	−11.2
	323 K	−11.8	−12.1	−11.6	−11.8
	∆*H*°, kJ/mol	11	11.6	7.4	8.1
	∆*S*°, J/mol·K	70	33	59	61.5
	*R* ^2^	0.93	0.88	0.98	0.78

*q_e, cal_* is the quantities of metal (mg/g) sorbed from the solution at equilibrium time, respectively, *k_2_* (g/mg·min) is the second-order rate constant, *q_m_* is maximum adsorption capacity (mg/g), *b* is Langmuir adsorption constant (L/mg), *R^2^* is coefficient of determination, Δ*G*° is Gibbs free energy change, Δ*H*° is enthalpy change, and Δ*S*° is entropy change.

**Table 4:** Biosorptive performance of different types of sorbents in zinc ions’ removal.

Sorbent	*q_max_*, mg/g	Concentrations Range, mg/L	pH	Reference
Mineral-organic sorbent	3.4–6.5	10–100	6.0	Present study
Activated carbon	103.8	0.005–0.025 mol/L	5.2	[6]
Yeast *Saccharomyces cerevisiae*	9–17	10–100	3.0–6.0	[19]
*Escherichia coli* biofilm supported on kaolin	78	10–200	5.0	[7]
*Staphylococcus epidermidis* supported on kaolin	49	10–200	5.0	[7]
Turkish leonardite-clinoptilolite mixture	454.5	20–400	6.0	[21]
Algerian bentonite	1.74	1–60	8.0	[28]
Zeolite	4.3	50	4.0	[15]

## Data Availability

Data is contained within the article or supplementary material.

## Supplementary Materials

The following are available online at https://www.mdpi.com/article/10.3390/ma14071760/s1, Figure S1: The Scanning electron microscope image of raw zeolite, Figure S2: Effect of time on efficiency of metal ions sorption on mineral-organic sorbent in ) Zn(II); b) Zn(II)-Sr(II)-Cu(II); c) Zn(II)-Ni(II)-Cu(II) and d) Zn(II)-Sr(II)-Cu(II)-Ba(II) systems, Figure S3: Effect of time on on efficiency of metal ions sorption on raw zeolite in ) Zn(II); b) Zn(II)-Sr(II)-Cu(II); c) Zn(II)-Ni(II)-Cu(II) and d) Zn(II)-Sr(II)-Cu(II)-Ba(II) systems, Figure S4: Kinetic curves describing metal ions sorption on mineral-organic sorbent in Zn(II)-Sr(II)-Cu(II) system, Figure S5: Kinetic curves describing metal ions sorption on mineral-organic sorbent in the Zn(II)-Ni(II)-Cu(II) system, Figure S6: Kinetic curves describing metal ions sorption on mineral-organic sorbent in the Zn(II)-Cu(II)-Sr(II)-Ba(II) system. Figure S7: Plot of lnK_d_ versus 1/T. Table S1: Kinetic parameters determined from the four applied models, Table S2: The parameters of the applied sorption isotherm models, Table S3: Thermodynamic parameters for metal sorption on mineral-organic sorbent.
