# Supplementary material for: Zinc-Containing Effluent Treatment Using Shewanella xiamenensis Biofilm Formed on Zeolite
Source: Materials (Basel). 2021 Apr 2;14(7):1760. doi: 10.3390/ma14071760 (PMC8038228; doi:10.3390/ma14071760)
Supplement: Supplementary file 1 [file materials-14-01760-s001.pdf]

materials

Supplementary

Zinc-Containing Effluent Treatment Using *Shewanella xiamenensis* Biofilm Formed on Zeolite

Inga Zinicovscaia [1,2,*], Nikita Yushin [1], Dmitrii Grozdov [1], Daler Abdusamadzoda [1], Alexey Safonov [3] and Elena Rodlovskaya [4]

1 Department of Nuclear Physics, Joint Institute for Nuclear Research, Joliot-Curie Str., 6, 1419890 Dubna, Russia; ynik_62@mail.ru (N.Y.); grozdov@jinr.ru (D.G.); martinez-91@mail.ru (D.A.)
2 Department of Nuclear Physics, Horia Hulubei National Institute for R&D in Physics and Nuclear Engineering, 30 Reactorului, MG-6, 077125 Bucharest-Magurele, Romania
3 Department of Biotechnology and Radioecology, Frumkin Institute of Physical Chemistry, Russian Academy of Science, 31 Leninsky Prospect, GSP-1, 119071 Moscow, Russia; alexeysafonof@gmail.com
4 Laboratory for Heterochain Polymers, A.N. Nesmeyanov Institute of Organoelement Compounds of Russian Academy of Sciences, Vavilova Str., 28, 119991 Moscow, Russia; ro745@mail.ru
* Correspondence: zinikovskaia@mail.ru; Tel.: +74-962-1656-09

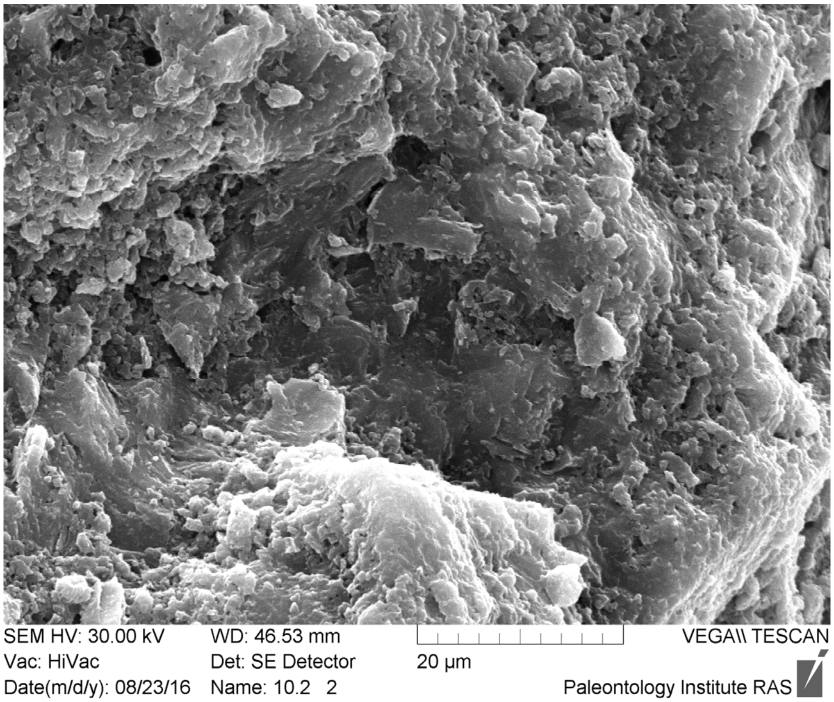

Figure S1. The Scanning electron microscope image of raw zeolite.

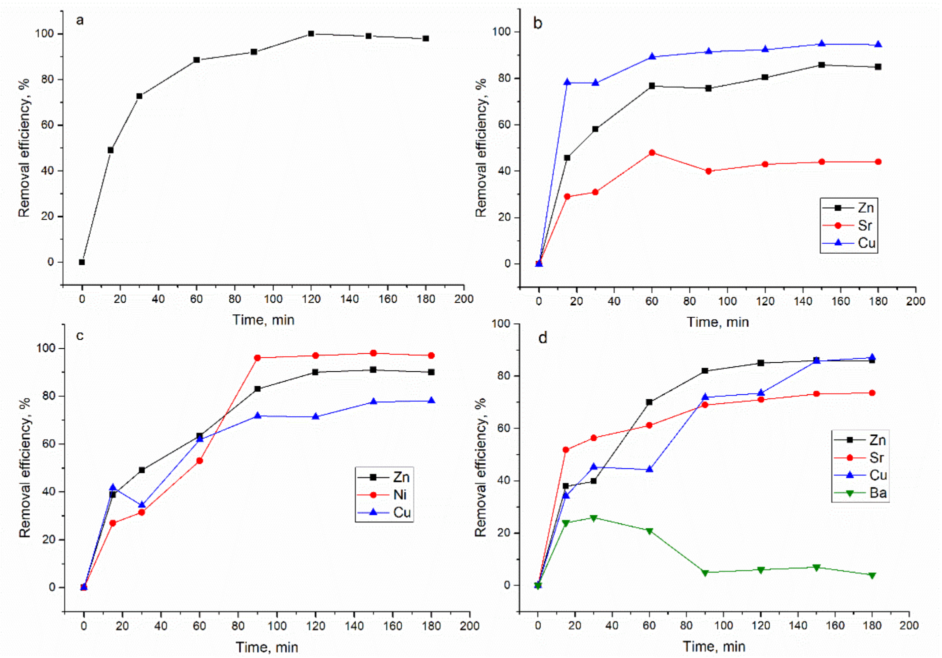

Figure S2. Effect of time on efficiency of metal ions sorption on mineral-organic sorbent in (**a**) Zn(II); (**b**) Zn(II)-Sr(II)-Cu(II); (**c**) Zn(II)-Ni(II)-Cu(II) and (**d**) Zn(II)-Sr(II)-Cu(II)-Ba(II) systems.

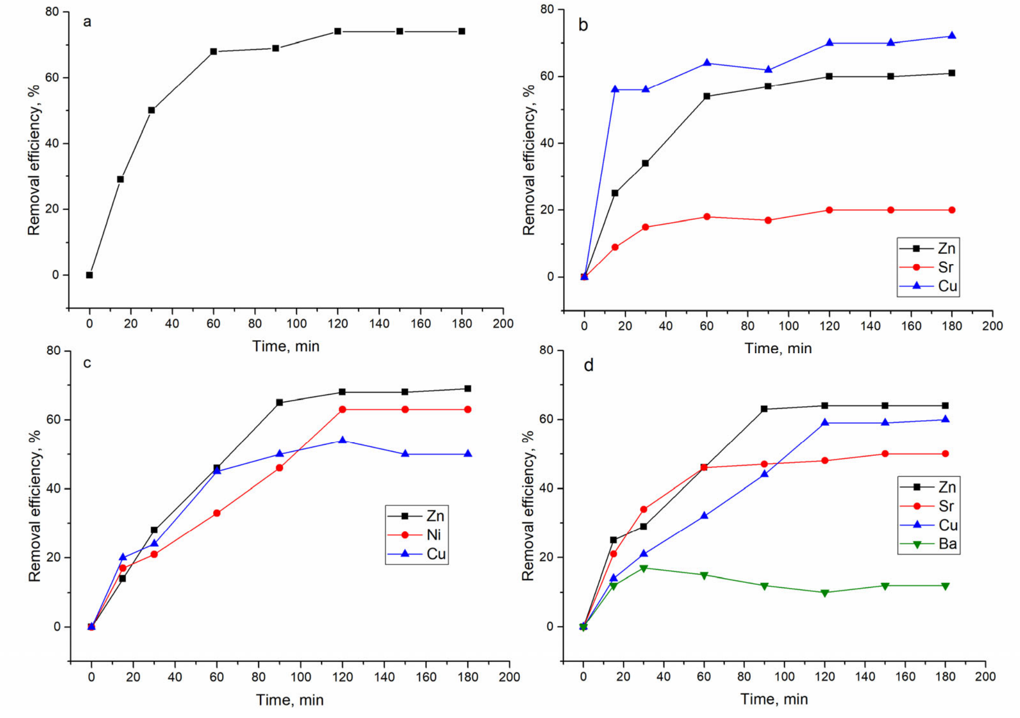

Figure S3. Effect of time on on efficiency of metal ions sorption on raw zeolite in (**a**) Zn(II); (**b**) Zn(II)-Sr(II)-Cu(II); (**c**) Zn(II)-Ni(II)-Cu(II) and (**d**) Zn(II)-Sr(II)-Cu(II)-Ba(II) systems.

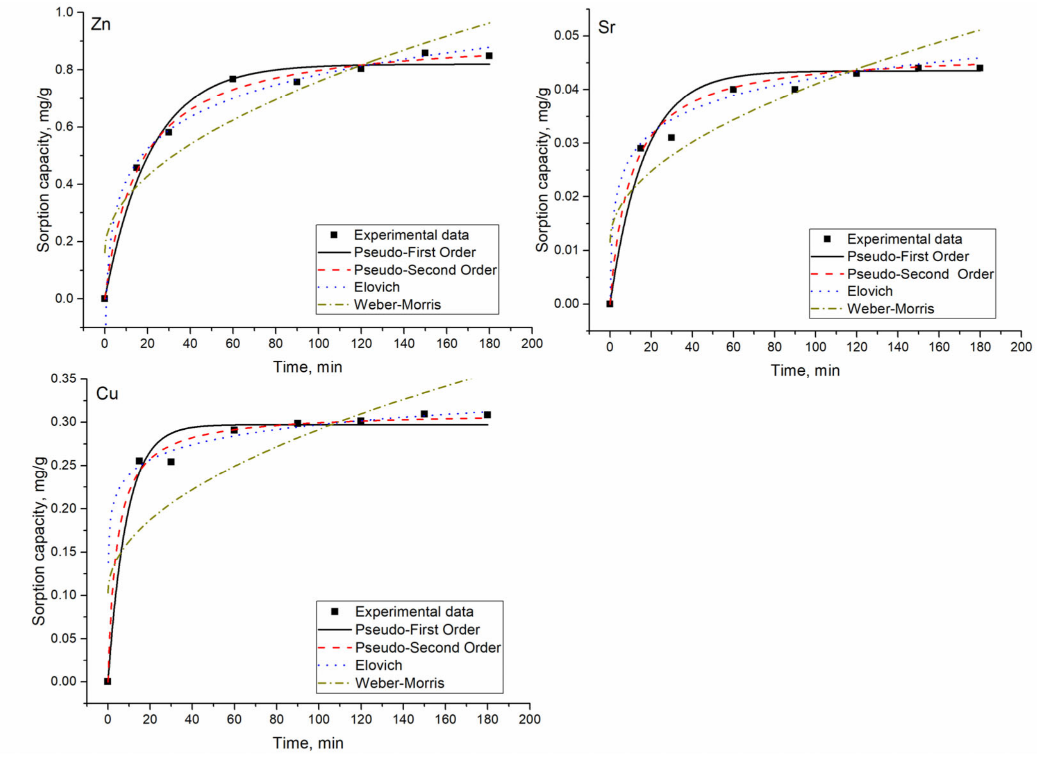

Figure S4. Kinetic curves describing metal ions sorption on mineral-organic sorbent in Zn(II)-Sr(II)-Cu(II) system.

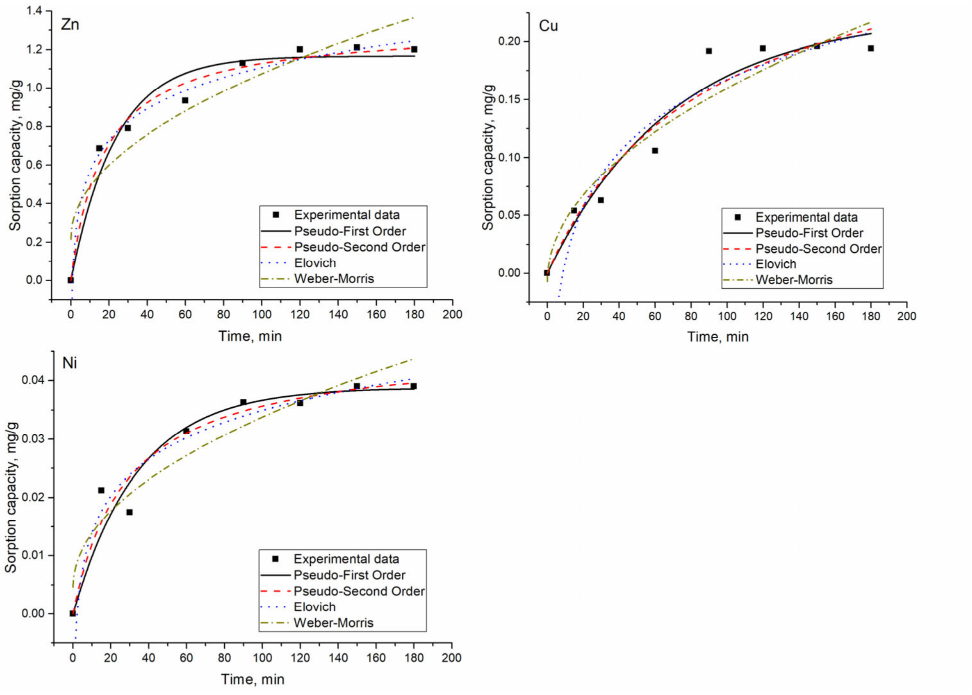

Figure S5. Kinetic curves describing metal ions sorption on mineral-organic sorbent in the Zn(II)-Ni(II)-Cu(II) system.

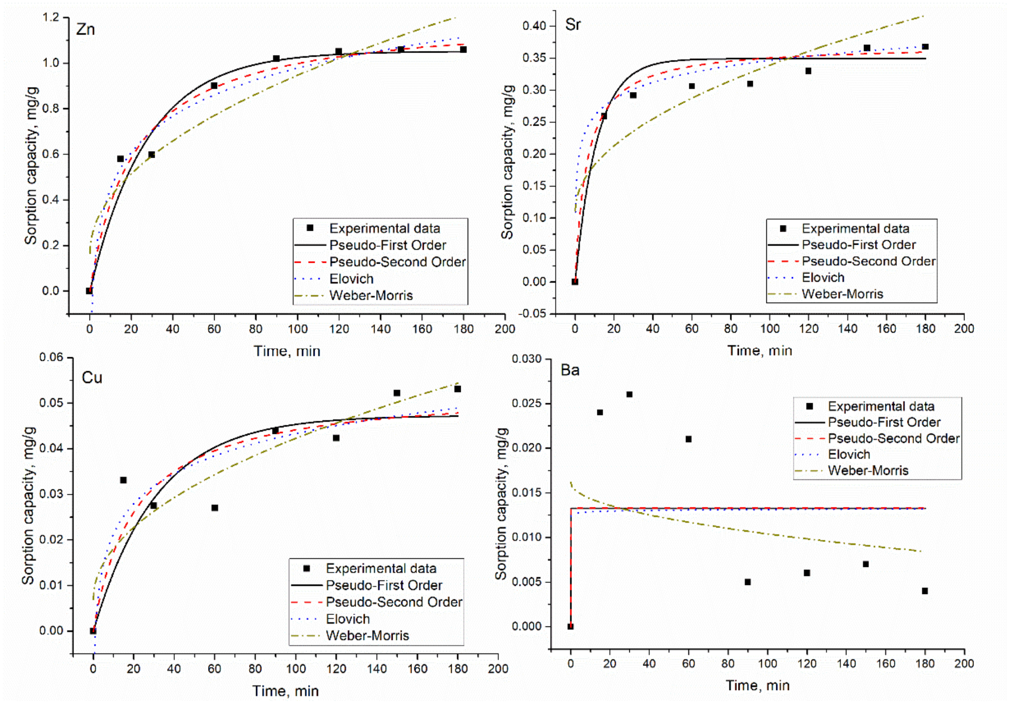

Figure S6. Kinetic curves describing metal ions sorption on mineral-organic sorbent in the Zn(II)-Cu(II)-Sr(II)-Ba(II) system.

Table S1. Kinetic parameters determined from the four applied models.

		**Zn(II)-Cu(II)-Sr(II)**		**Zi(II)-Ni(II)-Cu(II))**		**Zn(II)-Cu(II)-Sr(II)-Ba(II)**	
**Model**	**Metal**	**Cu**	**Sr**	**Ni**	**Cu**	**Cu**	**Sr**
	q_{exp}, mg/g	0.3	0.04	0.2	0.04	0.05	0.37
PFO	$q_{e,cal}$, mg/g	0.3	0.04	0.2	0.04	0.05	0.35
	k_1, min^{-1}	0.11	0.06	0.01	0.03	0.03	0.05
	R^2	0.98	0.94	0.95	0.92	0.73	0.96
PSO	$q_{e, cal}$, mg/g	0.3	0.05	0.3	0.05	0.05	0.37
	k_2, g/mg·min	0.6	2.8	0.7	4.7	0.9	0.4
	R^2	0.99	0.95	0.94	0.96	0.80	0.97
EM	α, mg/g·min	3.5	0.04	0.008	0.004	0.02	3.9
	β, g/min	39	155	14.5	109	104	27
	R^2	0.99	0.92	0.92	0.95	0.85	0.97
IPM	k_{diff}	0.02	0.003	0.01	0.002	0.003	0.02
	C_i	0.1	0.2	−0.007.	0.003	0.004	0.1
	R^2	0.63	0.7	0.9	0.9	0.75	0.65

PFO—pseudo-first-order models, PSO—pseudo-second-order model, EM—Elovich model, IMP—is Weber and Morris intraparticle diffusion model; q_{exp} and $q_{e,cal}$ are the quantities of metal (mg/g) sorbed from the solution at equilibrium and at t (min) time, respectively, and k_1 (1/min) is the pseudo-first-order rate constant, R^2 is coefficient of determination, k_2 (g/mg·min) is the second-order rate constant, α (g/mg·min) and β (g/mg) are the Elovich equation constants, k_{diff} is a rate parameter (mg/g·min$^{1/2}$), and C_i is the intercept, which relates to the thickness of the boundary layer.

According to coefficient of determination values, in Zn(II) system sorption of Zn(II) adjusts, preferably PFO and PSO models. In Zn(II)-Sr(II)-Cu(II) system sorption of Zn(II) and Cu(II) was better described by PSO and EM, while of Sr(II) by PSO. In Zn(II)-Ni(II)-Cu(II) system high coefficients of determination calculated for PSO and EM point at their relevance to describe the data for Zn(II) and Cu(II). The PFO better fits the data for Ni(II), indicating physical sorption. In Zn(II)-Cu(II)-Sr(II)-Ba(II) system the values of coefficient

of determination of PSO and EM for Zn(II), Cu(II) and Sr(II) were very close indicating their applicability for the description of experimental data. None of the models was applicable for the theoretical description of experimentally obtained clues for Ba(II).

Table S2. The parameters of the applied sorption isotherm models.

Model	Parameters	System			
		Zn(II)	Zn(II)-Cu(II)-Sr(II)	Zn(II)-Ni(II)-Cu(II)	Zn(II)-Cu(II)-Sr(II)-Ba(II)
Freundlich	K_F, mg/g	0.4	0.8	0.4	0.3
	1/n	0.52	0.3	0.4	0.4
	R^2	0.99	0.88	0.98	0.98
Temkin	a_T, L/g	0.2	0.8	0.3	0.3
	B, kJ/mol	1.7	3.5	2.8	3.1
	R^2	0.98	0.91	0.98	0.99

K_F and n are Freundlich equation constants, B manifests the sorption potential of the sorbent, a_T is Temkin constant, R^2 is coefficient of determination.

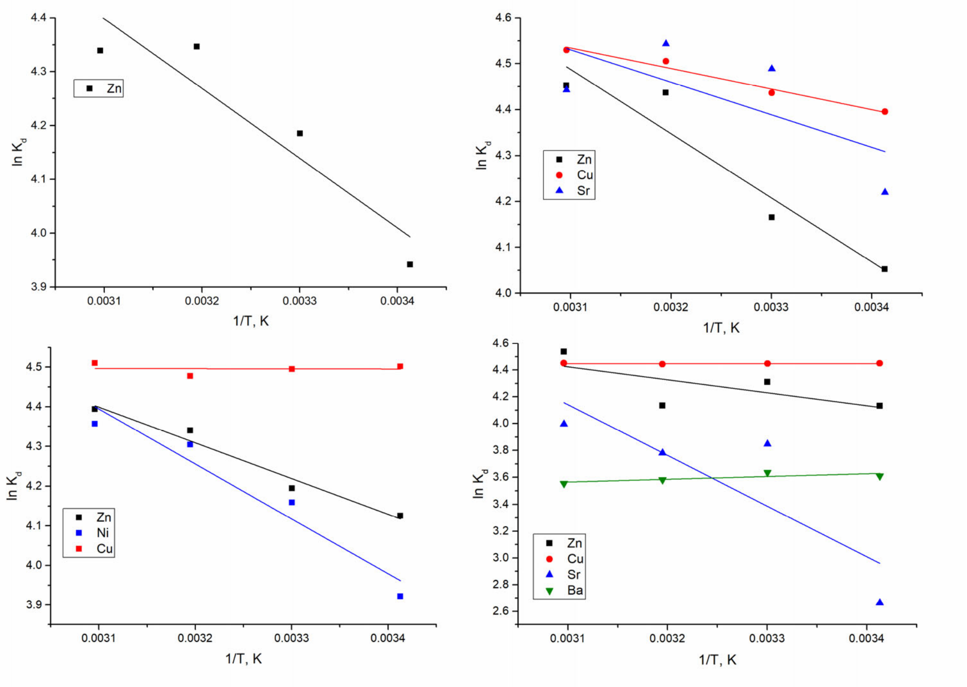

Figure S7. Plot of $\ln K_d$ versus 1/T.

Table S3. Thermodynamic parameters for metal sorption on mineral-organic sorbent.

System	Metal	$\Delta G°$, kJ/mol				$\Delta H°$, kJ/mol	$\Delta S°$, J/mol·K	R^2
		293 K	303 K	313 K	323 K			
Zn(II)-Cu(II)-Sr(II)	Cu	−10.6	−11.1	−11.6	−12.1	3.7	49	0.99
	Sr	−10.6	−11.2	−11.7	−12.3	5.9	56	0.7
Zn(II)-Ni(II)-Cu(II)	Ni	−9.7	−10.4	−11.1	−11.9	11	72	0.76
	Cu	−10.9	−11.3	−11.7	−12.1	0.03	33	0.96
Zn(II)-Cu(II)-Sr(II)-Ba(II)	Cu	−10.7	−11.1	−11.5	−11.8	−13	36.6	1.0
	Sr	−7.3	−8.7	−10.0	−11.3	31	132	0.80
	Ba	−8.8	−9.0	−9.3	−9.5	−1.7	24	0.88
